# Self-Reported History of Herpes Zoster and Awareness of Zoster Vaccination Among Healthcare Workers: A Multicenter Cross-Sectional Study

**DOI:** 10.3390/vaccines14080650

**Published:** 2026-07-24

**Authors:** Uğur Ergün, Selma Tosun, Ayşegül Seremet Keskin, Ebru Demiray Gürbüz, İrem Çiftci, Sinem Ayaz, İrem Aşkın Yılmaz, Şenol Çomoğlu, Işıl Deniz Alıravcı, Funda Şahin, Ahmet Şahin, Vahibe Aydın Sarıkaya, Özlem Türkmen Recen, Derya Seyman, Zeynep Türe Yüce, Beyza Arpacı Saylar, Emre Bayhan, Alev Çetin Duran, Ayşın Zeytinoğlu, Müge Toygar Deniz, Fatma Mutlu Sarıgüzel, Sevil Öztaş, Emine Çelik Tellioğlu, Ömür Mustafa Parkan, Zafer Adıgüzel, Rasih Felek, Ayşe İnci, Hasan Bozdağ, Şebnem Çalık, Ayşe Deniz Yüksel, Nagehan Didem Sarı, Rasih İmran Tan, İlyas Dökmetaş, Fatma Yılmaz Karadağ, Derya Öztürk Engin, Selçuk Kaya

**Affiliations:** 1 Department of Internal Medicine, Balıkesir Atatürk City Hospital, 10100 Balıkesir, Türkiye; 2 Department of Infectious Diseases and Clinical Microbiology, Faculty of Medicine, University of Health Sciences, 35100 İzmir, Türkiye; 3 Antalya Training and Research Hospital, University of Health Sciences, 07000 Antalya, Türkiye; 4 Department of Medical Microbiology, Faculty of Medicine, Dokuz Eylül University, 35100 İzmir, Türkiye; 5 Faculty of Medicine, Haliç University, 34000 İstanbul, Türkiye; 6 Ümraniye Training and Research Hospital, 34000 İstanbul, Türkiye; 7 Department of Infectious Diseases and Clinical Microbiology, Faculty of Medicine, Çanakkale Onsekiz Mart University, 17020 Çanakkale, Türkiye; 8 Department of Medical Microbiology, Faculty of Medicine, Adıyaman University, 02100 Adıyaman, Türkiye; 9 Department of Infectious Diseases and Clinical Microbiology, Faculty of Medicine, Dr. Ersin Arslan Training and Research Hospital, Gaziantep Islam Science and Technology University, 27000 Gaziantep, Türkiye; 10 Department of Infectious Diseases and Clinical Microbiology, Haydarpaşa Numune Training and Research Hospital, 34000 İstanbul, Türkiye; 11 Public Health Laboratory, Department of Medical Microbiology, İzmir Provincial Health Directorate, 35100 İzmir, Türkiye; 12 Eskişehir Private Gürlife Hospital, 26000 Eskişehir, Türkiye; 13 Department of Family Medicine, Faculty of Medicine, Başkent University, 06000 Ankara, Türkiye; 14 Department of Infectious Diseases and Clinical Microbiology, Adıyaman Training and Research Hospital, 02100 Adıyaman, Türkiye; 15 Microbiology Laboratory, Balıkesir University, 10100 Balıkesir, Türkiye; 16 Department of Medical Microbiology, Faculty of Medicine, İzmir University of Economics, 35100 İzmir, Türkiye; 17 Department of Infectious Diseases and Clinical Microbiology, Faculty of Medicine, Kocaeli University, 41000 Kocaeli, Türkiye; 18 Department of Medical Microbiology, Faculty of Medicine, Erciyes University, 38000 Kayseri, Türkiye; 19 Vocational School of Health Services, Karabük University, 78050 Karabük, Türkiye; 20 Department of Infectious Diseases and Clinical Microbiology, Fatih Sultan Mehmet Training and Research Hospital, University of Health Sciences, 34000 İstanbul, Türkiye; 21 Department of Microbiology, Faculty of Dentistry, Akdeniz University, 07000 Antalya, Türkiye; 22 Department of Infectious Diseases and Clinical Microbiology, Sultan Abdülhamid Han Training and Research Hospital, University of Health Sciences, 34000 İstanbul, Türkiye; 23 Department of Infectious Diseases and Clinical Microbiology, İstanbul Training and Research Hospital, University of Health Sciences, 34000 İstanbul, Türkiye; 24 Department of Infectious Diseases and Clinical Microbiology, Şişli Hamidiye Etfal Training and Research Hospital, University of Health Sciences, 34000 İstanbul, Türkiye; 25 Department of Infectious Diseases and Clinical Microbiology, Sancaktepe Şehit Prof. Dr. İlhan Varank Training and Research Hospital, University of Health Sciences, 34000 İstanbul, Türkiye; 26 Department of Medical Microbiology, Faculty of Medicine, İzmir Katip Çelebi University, 35100 İzmir, Türkiye

**Keywords:** herpes zoster, recombinant zoster vaccine, healthcare workers, awareness level

## Abstract

**Objective:** Varicella zoster virus (VZV), a member of the Herpesviridae family, is known as the causative agent of chickenpox (varicella). Following primary infection, VZV can remain latent for life and may reactivate over time to cause herpes zoster (shingles). Advanced age, immunodeficiency, and certain chronic illnesses are major risk factors for the development of herpes zoster. To prevent herpes zoster, a live zoster vaccine was licensed in 2006, followed by a recombinant zoster vaccine with higher efficacy in 2018. In Türkiye, the recombinant zoster vaccine was licensed in 2024. Raising awareness among healthcare workers is important both for their own health and the safety of vulnerable patients. This study aimed to evaluate healthcare workers’ knowledge and awareness of herpes zoster infection and zoster vaccines across the country, as well as to determine the prevalence of prior herpes zoster infection among them. **Methods:** After obtaining ethical approval, a nationwide multicenter survey was conducted. A questionnaire was distributed to healthcare workers via an online link. Participation was entirely voluntary. The survey collected demographic information (age, sex, profession, years of experience) and included questions on knowledge of herpes zoster, history of zoster infection, symptoms and severity (if applicable), awareness of the zoster vaccine, and vaccination attitudes. **Results:** A total of 5180 healthcare workers participated, of whom 3505 were female. The participants ranged in age from 18 to 79 years (mean age: 34.24 ± 11.52). Regarding professions: 47.9% were physicians, 21.3% were nurses or midwives, 0.8% were dentists or dental technicians, and 30% were from other healthcare professions. Among participants, 54.4% had ≤10 years of professional experience, 20% had comorbidities, and 10.5% were using immunosuppressive medications. It was found that 9.6% had previously had herpes zoster, 8.8% had no knowledge of the disease, 39.6% were aware of the vaccine, and 20.1% were unwilling to pay for vaccination. Multivariate binary logistic regression analysis revealed that being a physician significantly increased the likelihood of having correct knowledge by 1.961 times compared to other professions (*p* < 0.001, CI: 1.608–2.392). Similarly, those who had heard of herpes zoster were 3.191 times more likely to answer correctly than those who had not (*p* = 0.011, CI: 1.650–6.172). Male gender was significantly associated with a lower likelihood of having accurate knowledge compared to females (*p* = 0.001, OR = 0.720, CI: 0.594–0.874). **Conclusions:** This study revealed that healthcare workers’ knowledge and awareness regarding herpes zoster and its vaccines are generally insufficient. The recent licensing of the vaccine in Türkiye may explain this, but it is critical for healthcare professionals to be more informed about risk groups and vaccination indications in order to enhance the effectiveness of preventive healthcare services. Given the potential for serious complications of herpes zoster in elderly and immunocompromised individuals, the importance of vaccination should be emphasized more strongly to healthcare workers. Furthermore, the finding that 20.1% of those aware of the vaccine were deterred by its cost highlights the need to improve vaccine accessibility. Economic barriers preventing healthcare workers from being vaccinated emphasize the importance of making the vaccine widely available through public support to protect both healthcare workers and patient safety.

## 1. Introduction

Herpes zoster, commonly known as shingles, is a disease characterized by painful vesicular eruptions typically distributed along a single dermatome. The virus remains latent after the initial varicella infection and can reactivate, particularly in situations where the immune system is compromised, leading to the manifestation of the disease [1,2,3]. Herpes zoster is more frequently observed in elderly individuals, those undergoing immunosuppressive therapy, cancer patients, and organ transplant recipients. Advanced age, immunodeficiency, and certain chronic conditions are considered major risk factors for the development of herpes zoster [4,5].

The incidence of herpes zoster increases markedly in individuals aged 50 and above, and approximately half of those over the age of 80 are likely to experience the disease. One of the most significant complications of herpes zoster is postherpetic neuralgia (PHN), which is defined as persistent pain lasting more than 4–6 weeks following the resolution of the skin lesions. PHN is particularly common in the elderly [6,7,8,9]. To prevent herpes zoster, a live attenuated zoster vaccine was introduced in 2006 and recommended for individuals over the age of 65. However, the efficacy of this vaccine diminishes with advancing age. In 2018, a recombinant zoster vaccine with higher efficacy was licensed and is recommended in two doses for individuals aged 50 and older. This vaccine significantly reduces the risk of herpes zoster, especially in the elderly and immunocompromised individuals [10,11,12]. Although healthcare workers are not generally considered to be at higher risk of developing herpes zoster than the general population, they play a pivotal role in adult immunization programs through patient education, vaccine counseling, and evidence-based recommendations. Their knowledge and attitudes toward herpes zoster and its prevention may directly influence vaccine confidence and uptake among adults, particularly older individuals and those with immunocompromising conditions. Current international recommendations, including those from the Centers for Disease Control and Prevention and the World Health Organization emphasize herpes zoster vaccination for older adults and selected immunocompromised populations, highlighting the importance of healthcare professionals’ awareness of these recommendations. Therefore, assessing healthcare workers’ knowledge and awareness is essential for identifying educational needs and supporting effective implementation of adult vaccination strategies [13].

The aim of this study is to assess the knowledge and awareness levels of healthcare workers regarding herpes zoster infection, disease progression, and available vaccines. By conducting a large-scale, multicenter study with a sufficient sample size, we intend to provide data that can support improvements in both clinical practice and educational strategies nationwide.

## 2. Materials and Methods

This study was designed as a multicenter cross-sectional analytical study and was conducted among healthcare workers employed in various healthcare institutions across Türkiye. Data were collected via an online questionnaire. The questionnaire included items covering participants’ demographic information, knowledge levels regarding herpes zoster, history of prior herpes zoster infection, awareness of the vaccine, and attitudes toward vaccination. To ensure the validity and reliability of the survey, a pilot test was performed, and the questionnaire was reviewed by subject matter experts. Participation was voluntary, and anonymity was preserved throughout the study. Data obtained from the survey were analyzed using IBM SPSS Statistics version 24.

### 2.1. Healthcare System in Türkiye

Türkiye provides universal health coverage through the General Health Insurance (Genel Sağlık Sigortası, GSS) system, which ensures access to healthcare services for all citizens. The healthcare system is primarily financed through public funding and taxation, providing comprehensive healthcare services without a fee-for-service model for routine access within the public system. Preventive healthcare services, including the National Immunization Program, are provided free of charge. Older adults who meet national eligibility criteria are also offered recommended vaccines, such as the annual seasonal influenza vaccine, without cost through the public healthcare system. Healthcare professionals, including physicians and nurses, have the same healthcare coverage and benefits as other citizens under the GSS and do not receive additional healthcare privileges based on their profession. In Türkiye, private health insurance coverage remains limited, with approximately 2–3% of the population holding supplementary private health insurance.

### 2.2. Herpes Zoster Vaccine Availability in Türkiye

The recombinant herpes zoster vaccine available in Türkiye is Shingrix^®^, manufactured by GlaxoSmithKline (GSK, Brentford, UK). According to the manufacturer’s recommendations and current international immunization guidelines, the vaccine should be administered as a two-dose series to achieve optimal and durable protection, with an efficacy of approximately 90% against herpes zoster. During the study period, Shingrix was not included in the Turkish National Immunization Program and was therefore not provided free of charge by the public healthcare system. No official publicly available data exist regarding the number of herpes zoster vaccine doses distributed in Türkiye during 2024 or 2025, as the vaccine is not part of the routine national vaccination schedule and distribution data have not been publicly reported.

### 2.3. Study Design and Participants

This multicenter cross-sectional study was conducted between 20 November 2024 and September 2025 among healthcare workers from 24 healthcare centers representing major geographical regions of Türkiye, including the Aegean, Marmara, Central Anatolia, Black Sea, Eastern Anatolia, and Southeastern Anatolia regions. The questionnaire was distributed electronically via e-mail using institutional mailing lists. The study population consisted exclusively of clinical healthcare workers. Eligible participants included physicians, nurses, and other healthcare professionals who were actively involved in clinical practice and had direct patient contact. Participants were required to provide electronic informed consent before completing the questionnaire. Individuals who were retired, not actively practicing, had no direct clinical responsibilities, submitted incomplete questionnaires, or completed the survey more than once were excluded from the analysis. A total of 6200 healthcare workers were invited to participate, and 5180 completed the survey, yielding a response rate of 83.5%. A purposive sampling strategy was used to target healthcare professionals involved in the diagnosis, treatment, and prevention of herpes zoster, followed by convenience sampling based on voluntary participation through e-mail invitations. Because participants’ geographical locations and institutions were anonymized to preserve confidentiality, comparisons between individual regions or participating centers were not performed.

### 2.4. Statistical Analysis

Descriptive statistical methods, including frequency, percentage, mean, and standard deviation, were used to evaluate the demographic characteristics of the participants. Factors influencing unwillingness to pay for the zoster vaccine, the likelihood of possessing accurate knowledge, and the risk of not being vaccinated were analyzed using Multivariate Binary Logistic Regression Analysis. The evaluation of descriptive data was performed using IBM SPSS Statistics version 24.

## 3. Results

**Figure 1:**
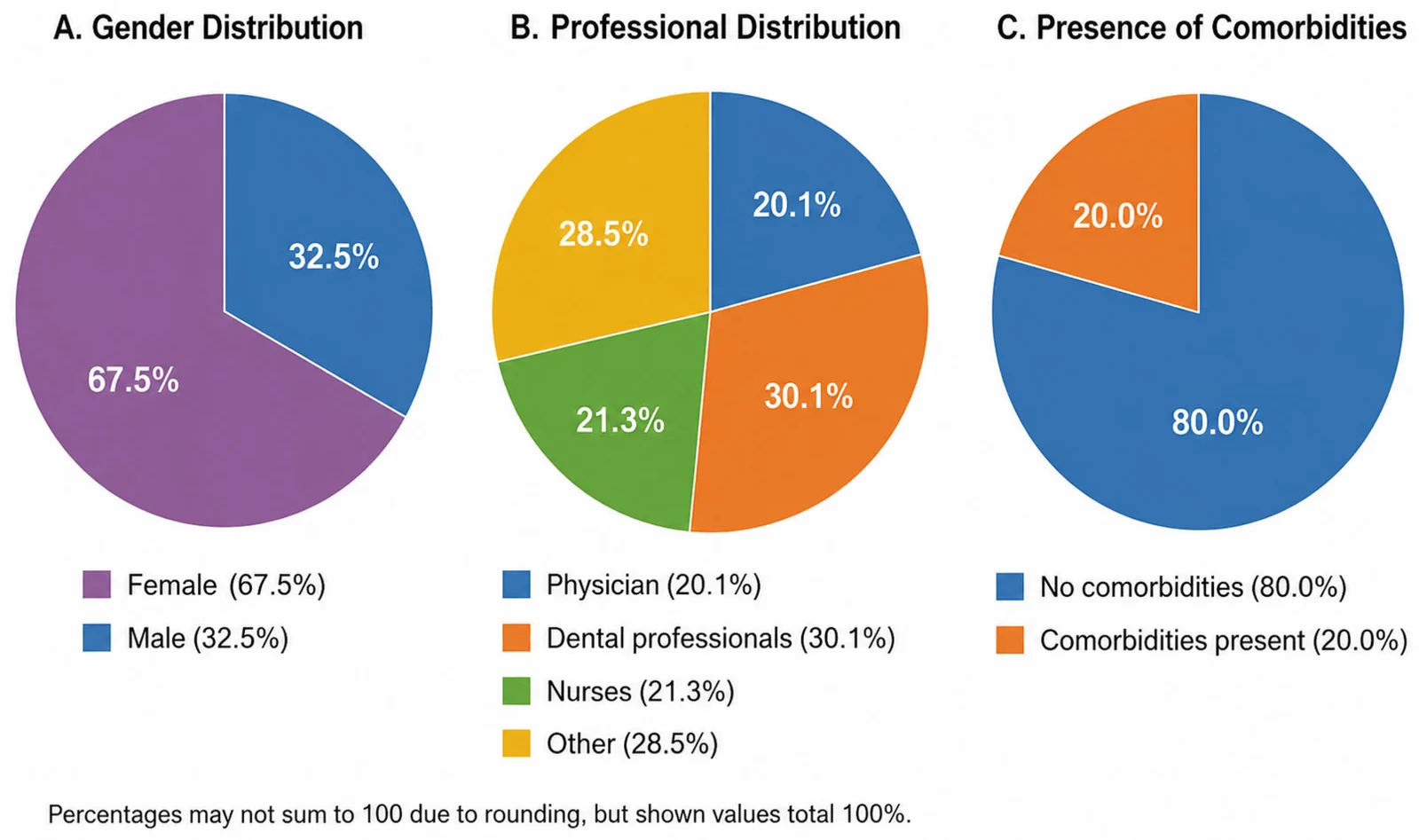
Demographic characteristics of the participants.

The mean age of the participants was 34.24 ± 11.52 years (Min = 18.00, Max = 79.00). Among the respondents, 2479 (47.9%) were physicians, 1104 (21.3%) were nurses or midwives, 39 (0.8%) were dentists or dental technicians, and 1558 (30.1%) belonged to other healthcare professions. It was found that 2817 participants (54.4%) had been working in the healthcare sector for 10 years or less, while the remaining had over 10 years of professional experience. A total of 1037 participants (20.0%) reported having a comorbid condition, and 543 (10.5%) stated that they were using immunosuppressive medication. The distribution of participants according to years of professional experience was relatively even, with 20% reporting the presence of a comorbid disease and 3% currently on immunosuppressive therapy (Figure 1).

A total of 498 participants (9.6%) reported having had herpes zoster. Only 457 participants (8.8%) stated that they had never heard of the disease, while 2050 (39.6%) reported being aware of the zoster vaccine, and 1041 (20.1%) indicated that they would not be willing to pay for the vaccine. Participants who had heard of the vaccine were asked a multiple-choice question to assess their knowledge level. Among them, 947 (46.2%) provided incorrect answers, while 1103 (53.8%) gave correct responses. Evaluation of responses to the herpes zoster-related knowledge questions revealed a generally high awareness of the disease itself; however, there were notable gaps in knowledge concerning symptoms, transmission routes, and complications.

According to the results of the Multivariate Binary Logistic Regression Analysis, being a physician significantly increased the likelihood of having accurate knowledge compared to other professions by 1.961 times (*p* < 0.001, CI: 1.608–2.392). Similarly, having previously heard of herpes zoster increased the odds of correct knowledge by 3.191 times compared to those who had not (*p* = 0.011, CI: 1.650–6.172).

Additionally, male participants were found to have a significantly lower likelihood of possessing accurate knowledge compared to female participants (*p* = 0.001, OR = 0.720, CI: 0.594–0.874) (Table 1).

When participants’ attitudes toward receiving the zoster vaccine were analyzed by years of professional experience, it was observed that those with fewer years in the profession were more inclined to get vaccinated. Specifically, 13% of those with less than 1 year of experience and 20% of those with 2–4 years of experience stated that they would get vaccinated. In contrast, this rate gradually decreased among those with 21 or more years of professional experience: only 10% of those with 31–40 years of experience and 2% of those with over 41 years of experience reported willingness to receive the vaccine. Similarly, the proportion of participants who stated “I will not get vaccinated” or “I am not in a risk group” increased with longer professional experience. Additionally, indecisive attitudes such as “I would get vaccinated if recommended by a doctor” and “I have concerns because the vaccine is new” showed a slight decrease as years in the profession increased (Table 2).

Participants’ responses to the question “Would you get the zoster vaccine for yourself?” showed statistically significant differences based on whether or not they had previously experienced herpes zoster. Among those who had a history of herpes zoster, 41.5% (n = 203) stated that they would get vaccinated, compared to 28.4% (n = 1323) among those who had not had the disease. In contrast, the proportion of participants who stated “I will not get vaccinated” was similar in both groups: 16.3% (n = 80) among those with prior herpes zoster and 15.7% (n = 739) among those without. The proportion of participants who believed they were not in a risk group was higher among those who had never had the disease (20.4% vs. 12%). Additionally, 18.4% (n = 90) of participants with prior herpes zoster and 21.3% (n = 997) of those without stated, “I would get vaccinated if recommended by a doctor.” The proportion of participants expressing hesitancy due to the vaccine being new was 11.8% (n = 58) among those with a history of herpes zoster and 14.4% (n = 675) among those without (Table 3).

Comorbidities included both chronic medical conditions and immunocompromising conditions relevant to herpes zoster risk. Immunocompromising conditions, including malignancy, chronic kidney disease, and other conditions associated with impaired immune function, were recorded as part of the participants’ medical history and included in the descriptive analyses.

9.2% of the participants (n = 490) reported having had shingles at least once in their lifetime. Among those who had experienced shingles, 74.3% were female (n = 364) and 25.7% were male (n = 126), with age ranges of 25–71 for women and 23–78 for men. Only 7 of these individuals required hospitalization due to a severe clinical presentation; the vast majority experienced the illness as outpatients with mild to moderate severity (Table 4).

Of the participants who had shingles, 66 (13.5%) had an additional illness or treatment that could cause immunosuppression. Among this group, 203 individuals (41.5%) stated that they would like to get vaccinated; 90 (18.4%) said they would do so if recommended by a doctor, 58 (11.8%) expressed hesitation due to the vaccine being new, 80 (16.3%) did not want to get vaccinated, and 59 (12%) did not consider themselves to be in a risk group.

Among healthcare workers who had previously experienced shingles, 16.3% (n = 80) stated that they did not want to receive the shingles vaccine. This rate was found to be 15.7% (n = 739) in the group without a history of shingles. Although no significant difference was observed between the two groups in terms of reluctance to get vaccinated, it was noted that hesitation or negative attitudes toward the vaccine persisted even among those who had experienced shingles.

The table illustrates the level of knowledge about the existence of the shingles vaccine among participants with and without a history of shingles. Notably, the proportion of participants unaware of the vaccine’s existence was high in both groups (Table 5).

When examining the price ranges that healthcare workers with and without a history of shingles would be willing to pay for the shingles vaccine, it was observed that a high proportion in both groups responded with “I have never thought about this” and “I would not pay anything.” This highlights a significant lack of awareness regarding the economic accessibility of the vaccine and a sensitivity to its cost (Table 6).

## 4. Discussion

This nationwide multicenter study evaluated healthcare workers’ knowledge of herpes zoster infection, its complications, and awareness of herpes zoster vaccination across multiple regions of Türkiye. The findings demonstrated that although healthcare workers generally possessed basic knowledge regarding herpes zoster, substantial deficiencies remained concerning disease transmission, complications, and particularly vaccine awareness. Most notably, only 39.6% of participants were aware of the existence of a herpes zoster vaccine. This finding indicates that knowledge regarding adult immunization against herpes zoster remains suboptimal even among healthcare professionals. Our findings are consistent with previous studies reporting inadequate awareness of herpes zoster vaccination among healthcare professionals, although awareness levels vary considerably across countries depending on national vaccination policies and educational strategies. In a previous study conducted among family physicians in Türkiye, 59.7% of physicians were aware of the herpes zoster vaccine, a considerably higher proportion than observed in the present study [14]. This discrepancy is likely attributable to differences in study populations, as the previous study included only physicians, whereas our study enrolled healthcare workers from multiple professional groups. Similarly, studies from Europe, North America, and Asia have demonstrated that awareness and recommendation rates are generally higher in countries where herpes zoster vaccination is incorporated into national adult immunization programs or where continuing professional education on adult vaccination is routinely provided [15,16,17]. Differences in healthcare systems, reimbursement policies, occupational vaccination programs, and cultural attitudes toward adult vaccination may therefore explain the variability observed across studies.

Although healthcare workers are not universally considered a high-risk population for herpes zoster, they play a pivotal role in adult immunization through patient education, vaccine counseling, and evidence-based recommendations. Consequently, inadequate awareness among healthcare professionals may negatively influence vaccine confidence and vaccine uptake in the general population. Improving healthcare workers’ knowledge regarding herpes zoster vaccination may therefore have benefits extending beyond individual protection by increasing public acceptance of adult immunization and strengthening preventive healthcare services. In the present study, although 39.6% of participants were aware of the herpes zoster vaccine, only 20.1% indicated that they would be willing to pay for vaccination. During the study period, individuals wishing to receive the herpes zoster vaccine outside the public reimbursement system were required to purchase it directly from community pharmacies. The retail cost of a single dose of Shingrix^®^ in Türkiye was approximately €157 (EUR) or USD180 (USD), representing a considerable financial burden for most adults and likely contributing to the low willingness to pay observed in our study. Previous studies have consistently demonstrated that vaccine cost is among the most important barriers to herpes zoster vaccination and substantially affects both vaccine accessibility and healthcare professionals’ willingness to recommend vaccination [18]. These findings emphasize the importance of reimbursement policies and improved vaccine accessibility for increasing adult vaccination coverage.

Our multivariable analysis further demonstrated that physicians were 1.894 times less likely to report willingness to receive herpes zoster vaccination than other healthcare professional groups. Although physicians generally possess greater medical knowledge, this finding suggests that knowledge alone may not translate into positive vaccination attitudes. Perceived personal susceptibility, vaccine accessibility, reimbursement policies, and misconceptions regarding vaccine indications may also influence vaccination decisions. Considering that physicians are among the most trusted sources of vaccine recommendations, improving their awareness and confidence regarding herpes zoster vaccination is particularly important for promoting vaccination acceptance among patients. Current international recommendations, including those of the Centers for Disease Control and Prevention and several professional societies, recommend recombinant herpes zoster vaccination for adults aged 50 years and older as well as immunocompromised adults aged 19 years and above. Although healthcare workers are not routinely identified as a priority vaccination group solely because of their occupation, their central role in counseling high-risk patients makes adequate knowledge of these recommendations essential. Continuous professional education focusing on adult immunization, workplace educational programs, and occupational health initiatives could substantially improve healthcare workers awareness and ultimately increase vaccination uptake among eligible populations. From a public health perspective, our findings suggest that occupational health services should incorporate regular educational interventions addressing adult vaccination, including herpes zoster vaccination [19,20,21,22,23]. Institutional continuing medical education activities, evidence-based educational materials, and workplace vaccination awareness campaigns may contribute to improving healthcare professionals’ knowledge and attitudes. Such interventions may subsequently enhance vaccine recommendations delivered during routine clinical practice and strengthen adult immunization programs. The present study has several strengths [24,25]. It is one of the largest nationwide multicenter studies evaluating healthcare workers’ awareness of herpes zoster vaccination in Türkiye and includes participants from multiple professional groups and healthcare institutions across different geographical regions. These characteristics increase the diversity of the study population and provide comprehensive evidence regarding healthcare workers’ knowledge and attitudes toward herpes zoster vaccination in a country where published data remain limited [26,27].

Nevertheless, several limitations should be acknowledged. First, the cross-sectional design precludes establishing causal relationships between participant characteristics and vaccine awareness. Second, participation was voluntary, introducing the possibility of selection bias. Third, herpes zoster history, vaccination status, and comorbidities were self-reported and therefore may be subject to recall bias or misclassification. Fourth, although participants were recruited from multiple regions of Türkiye, the sample may not fully represent all healthcare workers nationwide, limiting the generalizability of the findings. Finally, vaccination status and medical history were not verified through official medical records.

This study has several limitations that should be considered when interpreting the findings. First, because of its cross-sectional design, causal relationships between participant characteristics and herpes zoster vaccine awareness cannot be established. Second, participation was voluntary, which may have introduced selection bias, as healthcare workers with greater interest in vaccination may have been more likely to participate. Third, participants’ history of herpes zoster infection, vaccination status, comorbidities, and knowledge were based entirely on self-reported data, making the results susceptible to recall bias and reporting bias. Fourth, although the response rate was high, non-response bias cannot be completely excluded, as healthcare workers who did not participate may have differed in their knowledge or attitudes toward herpes zoster vaccination. Furthermore, although participants were recruited from 24 healthcare centers across several geographical regions of Türkiye, the study did not employ probability-based national sampling; therefore, the findings may not be fully generalizable to all healthcare workers in the country. Finally, participants’ vaccination status, medical history, and comorbid conditions were not verified using official medical records or immunization registries, which may have affected the accuracy of the reported information.

Future research should include prospective multicenter longitudinal studies evaluating changes in healthcare workers’ knowledge and attitudes over time, particularly following educational interventions or modifications to national vaccination policies. In addition, intervention studies assessing the effectiveness of structured educational programs and workplace vaccination campaigns would provide valuable evidence for improving herpes zoster vaccine awareness and acceptance among healthcare professionals. Qualitative studies exploring perceived barriers and facilitators to herpes zoster vaccination may further contribute to the development of targeted public health strategies.

## 5. Conclusions

In conclusion, this study highlights the need to increase awareness about herpes zoster and its vaccine among healthcare workers. To improve vaccination rates, it is essential to expand educational programs and awareness campaigns targeted at healthcare professionals. In this context, it is important for health authorities and academic institutions to develop more comprehensive strategies to address the knowledge gaps among healthcare workers.

## Figures and Tables

**Table 1:** Factors Affecting the Likelihood of Having Knowledge About the Zoster Vaccine.

	B	SH	Wald	df	*p*	OR	95% GA	
							AL	AL
Age	−0.003	0.007	0.192	1	0.661	0.997	0.983	1.011
Gender (Male)	−0.328	0.099	11.059	1	0.001	0.720	0.594	0.874
Profession (Physician)	0.673	0.101	44.230	1	<0.001	1.961	1.608	2.392
Years of experience (10 years or less)	−0.072	0.159	0.207	1	0.649	0.930	0.681	1.271
Comorbid condition (Present)	−0.023	0.122	0.035	1	0.851	0.977	0.770	1.240
Awareness of herpes zoster infection (Yes)	1.160	0.337	11.892	1	0.001	3.191	1.650	6.172
History of herpes zoster infection (Yes)	0.285	0.154	3.431	1	0.064	1.330	0.984	1.799

*N* = 2050, Nagelkerke R^2^ = 0.05, χ^2^ = 79.08, *p* < 0.001.

**Table 2:** Distribution of Willingness to Receive the Zoster Vaccine According to Years of Professional Experience.

Would You Get the Zoster Vaccine for Yourself?	Years of Professional Experience < 1 Years	2–4 Years	5–20 Years	11–20 Years	21–30 Years	31–40 Years	>41 Years	Total
**I will get vaccinated (n: 1526)**	200 (%13)	307 (%20)	291 (%19)	284 (%19)	266 (%17)	147 (%10)	31 (%2)	1526
**I will not get vaccinated (n: 819)**	72 (%9)	115 (%14)	157 (%20)	196 (%24)	200 (%24)	60 (%7)	19 (%2)	819
**I will get vaccinated if recommended by a doctor (n: 1037)**	170 (%16)	222 (%20)	203 (%19)	221 (%20)	190 (%17.5)	71 (%6.5)	10 (%1)	1087
**I am not currently in a risk group (n: 1015)**	126 (%12)	249 (%24.5)	259 (%25.5)	183 (%18)	139 (%14)	49 (%5)	10 (%1)	1015
**I have concerns because it is a new vaccine (n: 733)**	119 (%16)	148 (%20.5)	157 (%21.5)	146 (%20)	111 (%15)	44 (%6)	8 (%1)	733
**Total**	687	1041	1067	1030	906	371	78	5180

**Table 3:** Distribution of Participants’ Responses to the Question “Would You Get the Zoster Vaccine for Yourself?” According to Their History of Herpes Zoster Infection.

Would You Get the Shingles Vaccine for Yourself?	Those Who Have Had Shingles	Those Who Have Not Had Shingles
I would get vaccinated	203 (%41.5)	1323 (%28.42)
I would not get vaccinated	80 (%16.3)	739 (%15.7)
I am not in a risk group	59 (%12)	956 (%20.4)
I would get vaccinated if my doctor recommended it	90 (%18.4)	997 (%21.3)
I have concerns because the vaccine is new	58 (%11.8)	675 (%14.4)
Total	490	4690

**Table 4:** Clinical Characteristics of Participants Who Had Shingles.

Characteristic	Number (n)	Percentage (%)
Number of female participants (age range: 25–71)	364	%74.3
Number of male participants (age range: 23–78)	126	%25.7
Number of cases requiring hospitalization	7	-
Number of participants with immunosuppressive disease/treatment	66	%13.5

**Table 5:** Distribution of Knowledge About the Existence of the Shingles Vaccine According to Shingles History.

Knowledge About the Existence of the Shingles Vaccine	Those Who Have Had Shingles (n = 490)	Those Who Have Not Had Shingles (n = 4690)
Exists	205 (%42.0)	1843 (%39.3)
Does not exist	76 (%15.5)	807 (%17.2)
I don’t know	209 (%42.5)	2040 (%43.5)

**Table 6:** Distribution of Willingness to Pay for the Vaccine According to Shingles History.

How Much Would You Be Willing to Pay for the Vaccine?	Those Who Have Had Shingles	Those Who Have Not Had Shingles
<1000 TL	67 (%13.7)	595 (%12.7)
1100–2000 TL	36 (% 7.3)	255 (%5.4)
2100–3000 TL	22 (%4.5)	137 (%2.9)
3100–4000 TL	6 (%1.2)	45 (%0.9)
4100–5000 TL	3 (%0.6)	31 (%0.6)
>5100 TL	7 (%1.4)	76 (%1.6)
I have never thought about this	209 (%42.6)	2201 (%46.9)
I would not pay anything	84 (%17.1)	959 (%20.4)
The government should provide it free of charge	3 (%0.6)	34 (%0.7)
I would get vaccinated regardless of the cost	53 (%10.8)	357 (%7.6)
Total	490	4690

## Data Availability

The datasets generated and/or analyzed during the current study are not publicly available due to privacy and ethical restrictions but are available from the corresponding author on reasonable request and with permission from the relevant Ethics Committee.
